# Endurance running during late murine adolescence results in a stronger anterior cruciate ligament and flatter posterior tibial slopes compared to controls

**DOI:** 10.1186/s40634-021-00439-7

**Published:** 2022-01-03

**Authors:** Danielle N. Ochocki, Benjamin E. Loflin, Taeyong Ahn, Kaitlyn A. Colglazier, Andrew R. Young, Anna A. Snider, Elizabeth P. Bueckers, Edward M. Wojtys, Stephen H. Schlecht

**Affiliations:** 1 Department of Orthopaedic Surgery, Indiana University School of Medicine, VanNuys Medical Science Building, Room 0028, 635 Barnhill Drive, Indianapolis, IN 46202 USA; 2 Department of Biomedical Engineering, Indiana University Purdue University-Indianapolis, Indianapolis, IN USA; 3 Department of General Surgery, Indiana University School of Medicine, Indianapolis, IN USA; 4 Department of Orthopaedic Surgery, University of Michigan, Ann Arbor, MI USA

## Abstract

**Background:**

Anterior cruciate ligament (ACL) injury rates continue to rise among youth involved in recreational and competitive athletics, requiring a better understanding of how the knee structurally and mechanically responds to activity during musculoskeletal growth. Little is understood about how anatomical risk factors for ACL injury (e.g., small ACL size, narrow intercondylar notch, and steep posterior tibial slope) develop and respond to increased physical activity throughout growth. We hypothesized that the ACL-complex of mice engaged in moderate to strenuous physical activity (i.e., endurance running) throughout late adolescence and young adulthood would positively functionally adapt to repetitive load perturbations.

**Methods:**

Female C57BL6/J mice (8 weeks of age) were either provided free access to a standard cage wheel with added resistance (*n* = 18) or normal cage activity (*n* = 18), for a duration of 4 weeks. Daily distance ran, weekly body and food weights, and pre- and post-study body composition measures were recorded. At study completion, muscle weights, three-dimensional knee morphology, ACL cross-sectional area, and ACL mechanical properties of runners and nonrunners were quantified. Statistical comparisons between runners and nonrunners were assessed using a two-way analysis of variance and a Tukey multiple comparisons test, with body weight included as a covariate.

**Results:**

Runners had larger quadriceps (*p* = 0.02) and gastrocnemius (*p* = 0.05) muscles, but smaller hamstring (*p* = 0.05) muscles, compared to nonrunners. Though there was no significant difference in ACL size (*p* = 0.24), it was 13% stronger in runners (*p* = 0.03). Additionally, both the posterior medial and lateral tibial slopes were 1.2 to 2.2 degrees flatter than those of nonrunners (*p* < 0.01).

**Conclusions:**

Positive functional adaptations of the knee joint to moderate to strenuous exercise in inbred mice offers hope that that some anatomical risk factors for ACL injury may be reduced through habitual physical activity. However, confirmation that a similar response to loading occurs in humans is needed.

## Background

As anterior cruciate ligament (ACL) injury rates continue to rise among youth involved in recreational and competitive athletics, a better understanding of how the knee structurally and mechanically responds to activity during musculoskeletal growth is needed. To date, some of the primary clinical risk factors for ACL injury are anatomical in nature. These risk factors include a smaller ACL cross-sectional area [15, 26], a narrower intercondylar notch width/shape [24, 33], and a steeper posterior tibial plateau slope [6, 9]. Previously, it has been reported that in controlled animal studies whole bone and ACL size and strength can be positively influenced through both voluntary [29, 30] and forced [11, 20, 25, 35] endurance running. However, little is understood about how the knee in general, and the anatomical risk factors for an ACL injury specifically, are impacted by increased physical activity throughout musculoskeletal growth. Significant ACL-complex (i.e., ACL, entheses, and associated musculoskeletal structures) changes in pubescent mice following unrestricted voluntary cage wheel running across 6 to 10-weeks-of-age [30] have been reported. Notably, young C57BL/6 J (B6) mice that ran showed significantly (*p* < 0.01) lower intercondylar notch shape indices, a larger ACL cross-sectional area, and differential angular changes in the posterior tibial slopes, when compared to mice that did not run. These biomechanically induced differences in pubescent knee development were in accordance with an earlier mouse study [29] that reported significant differences in femoral diaphysis shape and strength following pre-pubescent exercise.

This study investigates whether these significant responses to increased physical activity in young mice continue throughout late adolescence and young adulthood or if plateaus occur beforehand as others have clinically suggested [27, 34]. We hypothesized that if work-to-run was increased in mice that ran throughout late adolescence into young adulthood, then their ACL-complexes would show greater functional adaptation to endurance running than that which we previously reported in younger mice [30].

## Methods

**Fig. 1:**
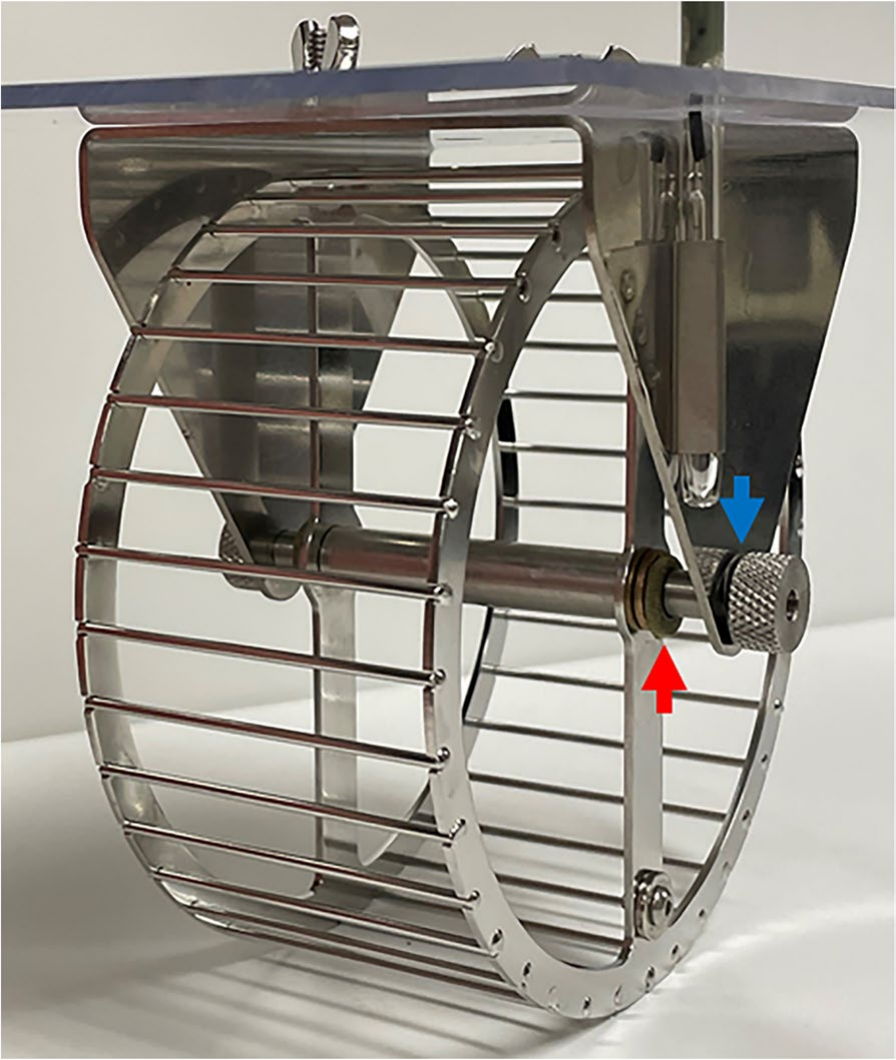
Resistive cage wheel setup. Red arrow points to Kevlar washer placed on the axle on both sides of the wheel to apply rotational resistance. Blue arrow point to Belleville disc spring used to maintain resistance during wheel rotation

With Institutional Animal Care and Use Committee approval thirty-six C57BL/6 J (B6) female inbred mice were bought from the Jackson Laboratory (Bar Harbor, ME) at 7 weeks of age and allowed to acclimate to 8 weeks of age (i.e., late adolescence). Mice were block-randomized into a control (*n* = 18) or exercise group (*n* = 18) by body weight (BW). All mice were individually housed for the duration of the study. Each mouse was provided with water and fed a standard rodent diet (D12450B; Research Diets, New Brunswick, NJ) ad libitum. Mice were kept on a 12-h light/dark cycle and supplied a nestlet for cage enrichment. B6 mice assigned to the exercise group (*n* = 18/strain) had free access (24 h/day) to a stainless-steel cage-wheel (115 mm outer diameter; Mini-Mitter Co., Inc., Murrysville, PA) for 4 weeks. The wheel axle had a 1.2 mm thick Kevlar washer (FTL180; Friction Technology Ltd., Caernarfon, Gwynedd, UK) placed on each side of the wheel with the selected resistance held stable using a Belleville disc spring. To keep equal resistance across all wheels, a 3.3 g weight was tied on the horizontal moment arm of the wheel. The collar of the axle was then tightened just enough to keep the resistance of the wheel via the disc spring (Fig. 1.). Frictional torque from each wheel was measured optically (Uno; Arduino LLC, Boston, MA, USA) by measuring deceleration and knowing the moment of inertia of the wheel. This measurement was recorded periodically throughout the study to keep frictional resistance on the same order of magnitude with an overall average of 4.85_E_-08. The resistance was chosen to induce the highest amount of work output without the mice disengaging with the wheel. Distance run (km) was calculated as the number of revolutions × the outer diameter of the wheel (mm) × π. Speed run (m/min) was calculated as the distance run divided by the user-defined measurement interval. Control mice were allowed normal cage activity during the study. BW and food weight were recorded one time per week throughout the 4-week experiment. Body composition measures (percent whole body and lean fat mass) were taken using an EchoMRI Body Composition Analyzer (EchoMRI, LLC, Houston, TX) just before and after study. All mice were euthanized at 12 weeks of age (i.e., early adulthood). From the left and right leg (*n* = 18/group/strain) of each mouse the *quadriceps, hamstrings, and gastrocnemius* muscle complexes, which collectively play a role in knee movement and stability, were carefully removed, weighed, and stored at − 20 °C in 1x phosphate-buffered saline (PBS) for a different study. The remaining left and right knees were also stored at − 20 °C in 1x PBS. Table 1 defines trait abbreviations frequently used throughout the manuscript.

**Table 1:** Frequently used abbreviations

*Body Measures*	
BW: Body weight	
LM: Lean mass	
FM: Fat mass	
*ACL Mechanics*	
UTS: Ultimate tensile strength	
S: Stiffness	
TYS: Tensile yield strength	
PYD: Post-yield displacement	
Work: Work-to-failure	
*Knee Morphology Measures*	
ANWI: Anterior intercondylar notch width index	
CNWI: Central intercondylar notch width index	
PNWI: Posterior intercondylar notch width index	
NSI: Intercondylar notch shape index	
Fem.Bicon.Wi: Femoral bicondylar width	
Not.Ht: Intercondylar notch height	
PMTS: Posterior medial tibial plateau slope	
PLTS: Posterior lateral tibial plateau slope	
ACL. Ell. CSA: ACL elliptical cross-sectional area	

### Mechanical properties

The right knees of both control and exercise mice were thawed at room temperature and dissected using a Leica S APO stereo microscope (Leica Microsystems, Inc., Buffalo Grove, IL). The patella, infrapatellar fat pad, both menisci, and the posterior cruciate ligament were removed, leaving the ACL and collateral ligaments intact. The medial and lateral collateral ligaments were left intact to prevent tibial rotation and support knee stability during potting. The tibial and femoral diaphyses were potted in custom brass tubing using a eutectic alloy (Cerrolow 174; Bolton Metal Products, Bellefonte, PA, USA). Once set, the potted knees were placed at 30° knee flexion in a custom loading fixture and affixed to a material testing system (Model 5944; Instron, Norwood, MA, USA). Thirty degrees of knee flexion is estimated to be the anatomical position of the rodent leg while standing [12]. Once the pots were secure, the lateral and medial collateral ligaments were cut and an acrylic hydration bath surrounding the fixture was filled with room temperature 1x PBS. For each potted knee, the following protocol was performed: 1) tensile preload applied to 0.02 N, 2) 10 pre-cycles between 0.02 N and 0.05 N at 0.5%/s, 3) a 300 s hold at 0.02 N, 4) ramp to 0.05 N at 5%/s followed by a 600 s hold, 5) return to zero-displacement and held for 60 s. Following this, a ramp-to-failure of the ACL was performed at a rate of 0.1%/s with data captured at 1000 Hz. Point of failure was assessed, with 100% of the failures occurring interstitially. Data analysis was performed with a custom MatLab (v2019b; The MathWorks, Inc., Natick, MA, USA) script. For each specimen, ultimate tensile strength (UTS; N), stiffness (S; N/mm), tensile yield strength (TYS; N), post-yield displacement (PYD; mm), and work-to-failure (Work; Nmm) were calculated.

### Knee joint morphology

Left knees of nonrunners and runners were thawed at room temperature and three-dimensionally imaged in 1x PBS at an 8 μm voxel size using nano-computed tomography (nanoCT) (nanotom-M, GE Sensing and Inspection Technologies, GmbH, Wunstorf, Germany). Imaging parameters were set to 90 kV, 375 mA, 1000 ms, 3 averages, and 1 skip, with a 0.3 mm aluminum filter. Grey values were converted to Hounsfield units using a calibration phantom having air, water, and a hydroxyapatite mimicker (1.69 mg/cc; Gammex, Middleton, WI, USA) as described previously [31].

**Fig. 2:**
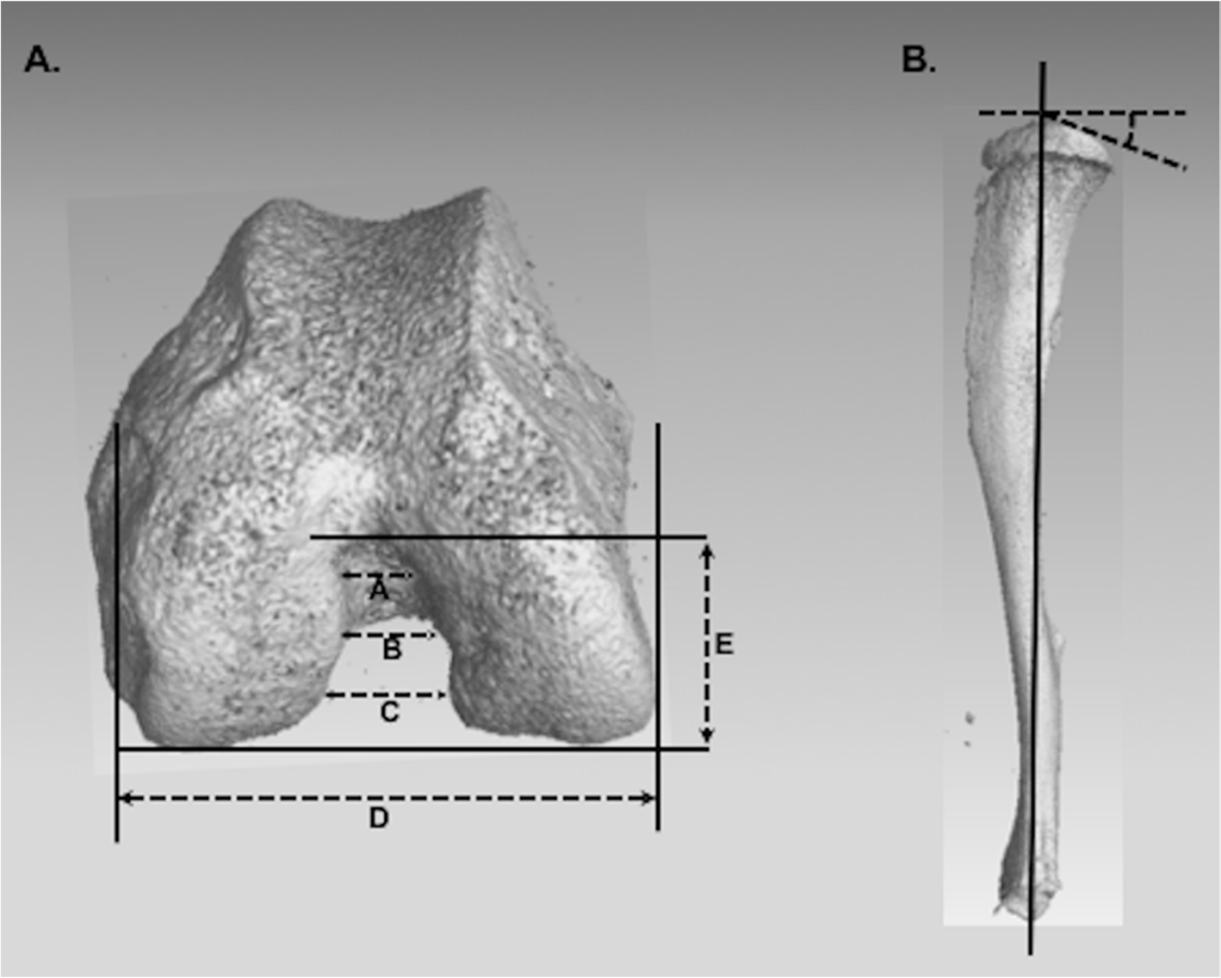
Femoral and tibial measurements. **A** 8 μm voxel image of distal femur demonstrating the intercondylar notch measurements (A. Ant.Not.Wi; B. Cen.Not.Wi; C. Post.Not.Wi; D. Fem.Bicon.Wi; E. Not.Ht). **B** 8 μm voxel image of proximal tibia demonstrating tibial plateau measurements. Solid line marks the longitudinal axis; dotted lines mark the perpendicular to the longitudinal axis and the angle of the posterior tibial slope from perpendicular. Reproduced with permission from [30]

Image analyses were conducted using Dragonfly software (v. 4.0, Object Research Systems, Montreal, Quebec). Analyses were in accordance with [30]. Briefly, femora were anatomically reoriented, and the slices based on morphological landmarks were used to directly measure the anterior (Ant.Not.Wi), central (Cen.Not.Wi), and posterior (Post.Not.Wi) intercondylar notch widths, intercondylar notch height (Not.Ht), and bicondylar width (Fem.Bicon.Wi). Variables derived from these measures included notch width indices (i.e., ANWI: Ant.Not.Wi/Fem.Bicon.Wi; CNWI: Cen.Not.Wi/Fem.Bicon.Wi; and PNWI: Post.Not.Wi/Fem.Bicon.Wi) and an estimate of the intercondylar notch shape (NSI: Cen.Not.Wi/Not.Ht) (Fig. 2A). Like the femora, tibiae were reoriented, and the slices used for analysis were defined based on morphological landmarks. For analyses, the longitudinal axis of the tibia was defined as the middle of the tibial intercondylar eminences through the center of the talar articular surface. A line perpendicular to this axis was then defined along the medial and lateral plateau surfaces. The angle between the posterior medial and lateral plateau surfaces and the perpendicular line was quantified, supplying the degree of posterior medial (PMTS) and posterior lateral (PLTS) tibial slope for each tibia (Fig. 2B).

### ACL morphology

After nanoCT imaging, the left knees of both runners and nonrunners were dissected using a Leica S APO stereo microscope equipped with a Leica DC3 digital color camera (Leica Microsystems, Inc., Buffalo Grove, IL, USA). The infrapatellar fat pad, collateral ligaments, menisci, and posterior cruciate ligament were carefully removed, leaving only the ACL intact. Using a custom holder, biplanar images of the ACL were taken in the coronal and sagittal planes to quantify the largest posterior length (ACL.Post.Le) and posterior and medial widths (ACL.Post.Wi and ACL.Med.Wi) of the ACL. Measurements were taken using ImageJ (NIH) to calculate ellipsoidal cross-sectional area (ACL Ell.CSA).

### Statistics

An a priori power analysis based on our previous studies estimated that for a power of 80% at a significance level alpha of 0.05, 12 mice per group were needed to detect differences. We included 18 mice per group to compensate for potential variability in ACL mechanical outcomes. All data were analyzed using Minitab v.20.3 (State College, PA, USA) and Prism v.9 (GraphPad Software, La Jolla, CA, USA). A Shapiro-Wilks test was conducted to test normality. All data was compared using a two-way analysis of variance (ANOVA) to test for a treatment main effect with body weight as a covariate, at a significance of *p* < 0.05. Tukey multiple comparisons test between exercised and control mice were assessed to find least square mean differences. Linear regression was used to look for both positive and negative associations between outcome measures and the average daily distance ran.

To test intraobserver reliability when selecting landmarks for the three-dimensional femoral and tibial morphology measurements, knees from a subset of mice were measured three separate times. Intraclass coefficient (ICC) values obtained from this test showed excellent reliability for all femoral measures (ICC: 0.938 – 0.996) and good reliability for all tibial measures (0.850 – 0.888).

## Results

### Endurance running resulted in larger muscles acting upon the knee

Table 2 includes the least square mean differences between runners and nonrunners for body composition. Following block randomization of all mice to treatment groups based on body weight (BW), weight distributions between runners and nonrunners were not significantly different (*p* = 0.81) at the beginning of the study. Following 4 weeks of voluntary physical activity, the runners weighed 3.42% (*p* = 0.01) more than the nonrunners. Additionally, runners consumed 20.5% more food over the 4 weeks compared to nonrunners. In terms of body composition, B6 runners began the study with significantly (*p* = 0.01) more lean mass (LM) and less fat mass (FM) than nonrunners, but by the end of the study LM and FM between runners and nonrunners were similar, with both treatment groups showing a reduction in LM and an increase in FM over the course of 4 weeks of late adolescent and early adulthood growth. Regarding muscle mass differences, runners showed significantly larger quadriceps (*p* = 0.02) and gastrocnemius (*p* = 0.05) muscles compared to nonrunners. Interestingly, they also showed a significantly smaller hamstring muscle (*p* = 0.05) (Table 3).

**Table 2:** Least square means differences in overall body composition between B6 runners and nonrunners

	Nonrunners	Runners	Difference (%)	*p*-value
**8wk BW (g)**	18.484 ± 0.71	18.542 ± 0.75	0.32	0.811
**12wk BW (g)**	20.267 ± 0.92	20.959 ± 0.67	3.42	**0.014**
**Difference (%)**	9.65	13.03		
***p*-value**	**< 0.001**	**< 0.001**		
**8wk LM (%)**	17.268 ± 0.85	18.429 ± 0.55	6.73	**0.001**
**12wk LM (%)**	14.227 ± 0.40	14.310 ± 0.60	0.58	0.632
**Difference (%)**	−17.61	−22.35		
***p*-value**	**< 0.001**	**< 0.001**		
**8wk FM (%)**	2.421 ± 0.31	2.120 ± 0.17	−12.43	**0.001**
**12wk FM (%)**	3.085 ± 0.45	3.243 ± 0.49	5.11	0.324
**Difference (%)**	27.46	52.99		
***p*-value**	**< 0.001**	**< 0.001**

**Table 3:** Least square means differences in primary muscles acting on the knee between B6 runners and nonrunners

	Nonrunners	Runners	Difference (%)	*p*-value
Quadriceps (g)	0.168 ± 0.01	0.179 ± 0.01	6.42	**0.021**
Gastrocnemius (g)	0.112 ± 0.01	0.121 ± 0.02	8.36	**0.053**
Hamstrings (g)	0.244 ± 0.03	0.220 ± 0.04	−9.86	**0.054**

### Significant knee trait differences related to a daily distance ran of at least 3 km/night

**Fig. 3:**
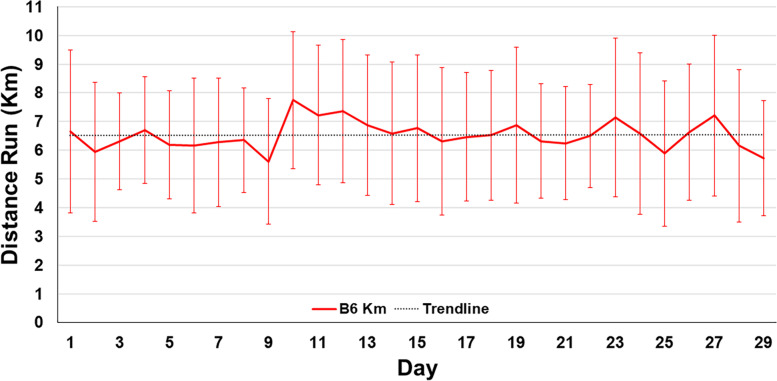
Daily average and standard deviations of daily kilometers run by all B6 mice in the exercise treatment group

B6 mice voluntarily ran a daily average of 6.53 km a night, with a range of 3.11 to 10.44 km across mice on a given night (Fig. 3) at an average speed of 435.28 m/hour/night. Outcome measures and statistical differences between treatment groups that are reported below showed no significant associations, via linear regression, with the amount a mouse of interest ran throughout the course of the study. All runners engaged with the wheel for at least 3 km/night on average (*n* = 18), resulting in ACL strength and knee morphology differences following running during late adolescences and early adulthood that were not present in nonrunners.

### Endurance running resulted in a stronger ACL

Table 4 highlights the ACL anatomical and mechanical outcomes between runners and nonrunners. Four weeks of voluntary, resistive cage wheel running throughout late adolescence and early adulthood did not result in a significantly larger ACL cross-sectional area (CSA) when compared to the nonrunners (*p* = 0.89). However, there was a significant difference in mechanical properties of the ACL between runners and nonrunners. At the completion of the study, runners collectively had an ACL that was stronger in tension than those that did not run. The ACL of runners had a 13% higher ultimate tensile strength (UTS) and a 16% higher tensile yield strength (TYS). These two traits contributed to a significantly higher (*p* = 0.04) work-to-failure (Work) compared to nonrunners. The ACL of runners was 6.9% stiffer (S) than that of nonrunners, though not significantly different. This increase in stiffness among the ACL of runners contributed to the higher Work and the nonsignificant 24% lower post-yield displacement (PYD). All ACLs failed interstitially (proximal third midsubstance).

**Table 4:** Least square means differences in ACL anatomical and mechanical properties between B6 runners and nonrunners

	Nonrunners	Runners	Difference (%)	*p*-value
CSA	0.070 ± 0.01	0.070 ± 0.01	0.79	0.892
UTS (N)	2.023 ± 0.25	2.283 ± 0.39	12.85	**0.028**
S (N/mm)	4.377 ± 0.56	4.678 ± 0.85	6.87	0.237
TYS (N)	1.825 ± 0.25	2.124 ± 0.45	16.39	**0.022**
PYD (mm)	0.283 ± 0.11	0.215 ± 0.13	−24.06	0.116
Work (Nmm)	0.868 ± 0.11	0.975 ± 0.17	12.32	**0.042**

### Endurance running resulted in flatter posterior tibial slopes

Table 5 highlights that B6 runners showed little difference in intercondylar notch width and shape measures, with only intercondylar notch height (Fem.Nt.Ht) being significantly 2% higher in runners compared to nonrunners. However, voluntary, resistive cage wheel running did have a significant effect on the posterior tibial slope when compared to nonrunners. Both posterior medial tibial slope (PMTS) and lateral tibial slope (PLTS) were significantly (*p* < 0.001) less steep among the runners.

**Table 5:** Least square mean differences in knee morphology measures between B6 runners and nonrunners

	Nonrunners	Runners	Difference (%)	*p*-value
***Fem. Traits***				
Fem.Bicon.Wi (mm)	2.678 ± 0.04	2.672 ± 0.04	−0.25	0.621
Fem.Nt.Ht (mm)	0.796 ± 0.02	0.812 ± 0.03	2.02	**0.039**
ANWI (mm/mm)	0.197 ± 0.01	0.189 ± 0.02	−4.07	0.114
CNWI (mm/mm)	0.284 ± 0.01	0.284 ± 0.01	0.24	0.806
PNWI (mm/mm)	0.290 ± 0.01	0.287 ± 0.01	−0.84	0.408
NSI (mm/mm)	0.955 ± 0.05	0.936 ± 0.05	−1.97	0.228
***Tib. Traits***				
PMTS (°)	25.220 ± 1.06	23.977 ± 0.96	−4.93	**0.001**
PLTS (°)	27.445 ± 0.82	25.292 ± 0.58	−7.84	**0.001**

## Discussion

The data from this study supports the hypothesis that resistive voluntary cage wheel running throughout late adolescence and early adulthood positively affects ACL-complex mechanics and knee structure in female mice. Mice that ran showed significantly larger muscle complexes acting upon the knee (i.e., quadriceps and gastrocnemius), greater ACL tensile strength, and a flatter posterior tibial plateau. Confirmation of the hypothesis suggests that the murine ACL-complex can functionally adapt to repetitive load perturbations up to at least early adulthood, which would agree with both murine and human studies across other musculoskeletal tissues (diaphyseal bone [3, 29], muscle [23, 28], tendon [8, 17]) that found a positive morphological and/or strength effect in response to loading. If translatable to humans, these findings are potentially clinically important for ongoing efforts towards reducing anatomical risk factors for ACL injury, since the ACL-complex traits that showed the greatest differences following running compared to nonrunners in this study are all well-established risk factors for ACL injury among high school and collegiate aged athletes [6, 7, 15, 22].

Along with a flattening of the posterior tibial slopes, the most significant differences between runners and nonrunners were the ACL mechanical properties. Runners showed a significantly stronger ACL compared to nonrunners, which concurs with earlier findings in adolescent rabbits [35] and rats [11] that were trained for endurance running on treadmills. Interestingly, higher tensile strength does not appear to be related to the size of the ACL in our mice since the cross-sectional areas in runners were similar to those in nonrunners. In earlier animal [30] and clinical [4] work it was hypothesized that increased load perturbations that drove a larger ACL size during adolescence was indicative of a stronger ACL. Based on the findings of our current study, the greater ACL strength in late adolescent runners may be attributable to collagen fibril and/or extracellular matrix differences between treatment groups. Current work in our lab is investigating the contribution of nano- and micro-scopic structural and compositional properties following an exercise perturbation on whole ACL strength. The lack of a significant difference in ACL size between treatment groups may potentially be related to the size limiting effects of the intercondylar notch, which is more stenotic (A-shaped) in runners and nonrunners. Recently, Barnum et al. [2] and Bouras et al. [10] found an association between a stenotic intercondylar notch and ACL injury risk in female patients, which potentially reflects a greater risk for ACL impingement while transitioning from knee flexion into extension. Interestingly, in earlier running studies using pubescent inbred strains of mice (i.e., B6 and A/J), the ACL of B6 runners was found to be 13.5% larger than that of nonrunners, while A/J runners showed a 7% smaller ACL than their respective controls. The authors attributed this to the intercondylar notch shape differences between B6 and A/J runners, with the former having a wider, more parabolic intercondylar notch, and the latter having a significantly narrower, more stenotic intercondylar notch compared to their respective controls. The authors hypothesized that in these young mice the ACL and surrounding intercondylar notch were potentially working in concert (i.e., canalized [31]) to balance ACL growth with that of its osseous compartment [30].

Though there were no major intercondylar notch differences between runners and nonrunners, there were significant differences across the posterior slopes of the tibial plateau. Similar to what was observed in younger exercising B6 mice, the medial and lateral posterior tibial slopes were significantly 5 - 8% less steep following 4 weeks of resistance running in older B6 mice [30]. On average, the PLTS was 2.2° flatter and the PMTS was more than 1.2° flatter among mice that ran. If this degree of change resulting from increased physical activity can also occur in young active humans, this would offer hope towards reducing one of the most critical anatomical risk factors for ACL injury. Surprisingly, it has been prospectively demonstrated that a one degree increase in PLTS can impart a 21.7% increase in noncontact ACL injury risk among high school and collegiate female athletes [6]. If increased physical activity during this critical growth period can reduce the PLTS by two degrees, this could have a huge clinical impact on reducing noncontact ACL injuries. However, the challenge likely remains to get children to engage in moderate to strenuous activity on a habitual basis early in musculoskeletal development. Increasingly, national and international organizations have recommended that children and adolescents (5-17 years of age) accumulate at least 60 min of moderate to strenuous activity daily to combat poor health outcomes associated with a sedentary lifestyle [1, 14, 36]. The trends in sedentary behavior among this age group are alarming with only 27% of high school students accumulating any level of physical activity for at least 60 min/day, and 41% and 32% of students playing video games and watching television for 3 or more hours a day, respectively. Based on our findings, if we continue to promote and encourage early habitual participation in moderate to strenuous physical activity, then not only will this improve muscle [8, 17, 23] and bone strengthening [3, 29] but may also provide additional benefits for reducing knee injuries among young individuals via positive knee structure and mechanical modifications. This is particularly important considering that the steepness of the posterior tibial slope directly relates to the magnitude of anterior tibial shear force generated from compressive loading (e.g., weight bearing) [21]. Thus, larger shear forces can result in greater anterior tibial translation [19] and greater ACL force [32], increasing the tendency for the ACL to be overloaded. Engaging in daily moderate to strenuous physical activity (e.g., endurance running) early on will increase compressive forces experienced by the knee, and may stimulate nonlinear bone remodeling responses to mechanical strain across the tibial plateau during growth to both strengthen the bone and shape it for load predictability without forfeiting knee stability [5].

There are many limitations to this study that must be considered when interpreting the results. First, differences in ACL-complex traits between runners and nonrunners were only quantified at the end of the study. Therefore, how these differences emerged between 8 to 12 weeks in these mice could not be assessed since there were no baseline or mid-study measures of these traits. Presumably, since the B6 runners and nonrunners are inbred siblings with a low degree of genetic variability [13], the observed mechanical and morphologic differences between groups are likely most attributable to the difference in activity between groups. Nevertheless, we cannot confidently confirm our perspective without longitudinally tracking what influence physical activity has on the musculoskeletal growth trajectory of these mice. Second, the resulting differential phenotypes between those that ran and those that did not were only demonstrated in female mice. Therefore, it is unknown whether male B6 mice, which have been shown by others to voluntarily run less on a daily basis [16], would show similar differences following exercise during late adolescence. Third, the lifespan of mice is much shorter than that of humans, which equates in a pubescence growth phase of ~ 4 – 5 weeks in inbred mice compared to ~ 4 – 5 years in humans [18]. Therefore, direct age comparisons cannot be made. However, direct comparisons in terms of biological changes during major developmental phases can be made, which is the perspective through which our interpretations of the data are based. Lastly are the locomotor and biomechanical differences between mice, and for that matter all commonly used animal models, compared to humans. Though mice are quadrupedal, previous research concerning voluntary cage wheel running among B6 mice showed consistent diaphyseal changes in bone morphology between the femora and humeri [29], suggesting that functional responses to increased physical activity via a cage wheel is largely shared amongst all four limbs. Moreover, mice have a very similar knee structure to that of humans [12] that should result in roughly similar knee kinematics during straight-line endurance running.

## Conclusions

Despite these study limitations, we confirmed the hypothesis that mice participating in resistive, voluntary cage wheel running during late adolescence and early adulthood would show significant mechanical and morphological differences in knee joint structures compared those that did not run. This study demonstrates that commonly perceived non-modifiable risk factors for ACL injury are indeed modifiable through increased physical activity during musculoskeletal growth in inbred mice. However, how this translates to humans is unknown. Therefore, we conclude that if a similar adaptive response is present in humans, then it may be possible to drive positive mechanical and structural changes in anatomical risk factors for ACL injury via moderate to strenuous physical activity throughout musculoskeletal growth. This would offer hope to players, parents, coaches, trainers and clinicians that aspects of knee joint anatomy that may be high risk in some individuals for an ACL injury, can be potentially modified over time to reduce the likelihood of injury while also potentially improving knee kinematics overall.

## Data Availability

The data generated and/or analyzed during the current study are available from the corresponding author on reasonable request.
